# Pharmacologic activity and pharmacokinetics of metabolites of regorafenib in preclinical models

**DOI:** 10.1002/cam4.883

**Published:** 2016-10-13

**Authors:** Dieter Zopf, Iduna Fichtner, Ajay Bhargava, Wolfram Steinke, Karl‐Heinz Thierauch, Konstanze Diefenbach, Scott Wilhelm, Frank‐Thorsten Hafner, Michael Gerisch

**Affiliations:** ^1^ Drug DiscoveryBayer Pharma AGMüllerstraße 178Berlin13353Germany; ^2^ Experimental Pharmacology & Oncology GmbHRobert‐Rössle‐Str. 10Berlin‐Buch13125Germany; ^3^ Shakti Bioresearch1 Bradley Road, STE 401WoodbridgeConnecticut06525; ^4^ Pharmacokinetic Imaging Consulting & Autoradiography ServicesHalverscheid 13, D‐58553HalverGermany; ^5^ Bayer HealthCare Pharmaceuticals100 Bayer BlvdWhippanyNew Jersey07981

**Keywords:** Antitumor activity, metabolite, multikinase inhibitor, pharmacology, regorafenib

## Abstract

Regorafenib is an orally administered inhibitor of protein kinases involved in tumor angiogenesis, oncogenesis, and maintenance of the tumor microenvironment. Phase III studies showed that regorafenib has efficacy in patients with advanced gastrointestinal stromal tumors or treatment‐refractory metastatic colorectal cancer. In clinical studies, steady‐state exposure to the M‐2 and M‐5 metabolites of regorafenib was similar to that of the parent drug; however, the contribution of these metabolites to the overall observed clinical activity of regorafenib cannot be investigated in clinical trials. Therefore, we assessed the pharmacokinetics and pharmacodynamics of regorafenib, M‐2, and M‐5 in vitro and in murine xenograft models. M‐2 and M‐5 showed similar kinase inhibition profiles and comparable potency to regorafenib in a competitive binding assay. Inhibition of key target kinases by all three compounds was confirmed in cell‐based assays. In murine xenograft models, oral regorafenib, M‐2, and M‐5 significantly inhibited tumor growth versus controls. Total peak plasma drug concentrations and exposure to M‐2 and M‐5 in mice after repeated oral dosing with regorafenib 10 mg/kg/day were comparable to those in humans. In vitro studies showed high binding of regorafenib, M‐2, and M‐5 to plasma proteins, with unbound fractions of ~0.6%, ~0.9%, and ~0.4%, respectively, in murine plasma and ~0.5%, ~0.2%, and ~0.05%, respectively, in human plasma. Estimated free plasma concentrations of regorafenib and M‐2, but not M‐5, exceeded the IC_50_ at human and murine VEGFR2, suggesting that regorafenib and M‐2 are the primary contributors to the pharmacologic activity of regorafenib in vivo.

## Introduction

Regorafenib is an oral multikinase inhibitor that blocks the activity of multiple protein kinases, including those involved in the regulation of tumor angiogenesis (VEGFR1, 2, and 3, and TIE‐2), oncogenesis (KIT, RET, RAF‐1, BRAF, and BRAF^V600E^), and the tumor microenvironment (PDGFR and FGFR) 1, 2, 3. Early‐phase clinical studies of regorafenib demonstrated antitumor activity in a range of solid tumors 2, 3, 4, 5, 6, which has been shown to translate into clinical benefit in phase III trials in treatment‐refractory metastatic CRC and GIST 7, 8, 9. Results of the phase III trials have led to regulatory approval for the use of regorafenib in metastatic CRC and GIST in a number of countries globally.

As regorafenib is administered orally, characterization of its biotransformation pathways and metabolite activity is of particular importance. In humans, regorafenib is primarily metabolized in the liver by oxidative and conjugative biotransformation, leading to the formation of the primary N‐oxide metabolite M‐2, which then enters systemic circulation. Further oxidative biotransformation results in the demethylated N‐oxide derivative M‐5, a secondary metabolite of regorafenib (Fig. S1) 10. Clinical trials in patients with advanced cancer demonstrated that total plasma exposure to each of the metabolites, M‐2 and M‐5, at steady state was comparable to that of the parent compound after administration of regorafenib 160 mg once daily at the approved 3 weeks on/1 week off dosing regimen 4, 5.

Given the substantial systemic exposure to the M‐2 and M‐5 metabolites observed in clinical trials, we investigated the pharmacokinetics and pharmacodynamics of regorafenib and these two major human metabolites in vitro and in an in vivo murine model in order to assess their potential contribution to the overall pharmacologic activity of regorafenib in humans.

## Methods

### Cell lines and reagents

HuVECs and HuLECs were purchased from Lonza (Walkersville, MD). The human breast cancer cell line MDA‐MB‐231 (K‐RAS^G13D^, BRAF^G464V^) and the human CRC cell line HT‐29 (BRAF^V600E^) were purchased from the American Type Culture Collection (LGC Standards GmbH, Wesel, Germany).

Recombinant human VEGF‐A, VEGF‐C, FGF7, and anti‐FGFR2 antibody (MAB6842) were purchased from R&D (Minneapolis, MN). Antibodies against pAKT (#3787; pS473), AKT (#4691), pERK1/2 (#9106; pT202/pY204), ERK1/2 (#9107), pVEGFR2 (#2478; pY1175), and VEGFR2 (#2479) were from Cell Signaling Technologies (Danvers, MA); pVEGFR3 (CB5793; pY1063/pY1068) was from Cell Applications (San Diego, CA); anti‐pFlg antibody (sc‐30262R; pY653/654) was from Santa Cruz Biotechnologies (Dallas, TX), and anti‐VEGFR3 antibody (MAB3757) was from Millipore (Billerica, MA).

Regorafenib and its metabolites M‐2 and M‐5, both cold and radio‐labeled, were obtained from Bayer Pharma R&D (Wuppertal, Germany). They were dissolved in 100% dimethyl sulfoxide for in vitro applications and in polypropylene glycol/polyethylene glycol 400/Pluronic F68 (42.5 g/42.5 g/15 g) + 20% aqua for in vivo tumor growth inhibition and pharmacokinetic studies.

Analyte concentrations were measured by high‐performance liquid chromatography using a Hewlett Packard 1100 Series system (Palo Alto, CA), with tandem mass spectrometric detection using an Applied Biosystems API 4000 (Thermo Fisher Scientific, Waltham, MA). The assay was validated according to the applicable guideline on method validation 11. The lower limit of quantitation for the assay was 2.00 μg/L, with a linear range of 2.00–20,000 *μ*g/L using a sample volume of 100 *μ*L.

### Kinome selectivity profile analysis

The kinome‐wide selectivity profiles of regorafenib, M‐2, and M‐5 were analyzed using an active‐site competitive binding assay as previously described 12 (KINOME*scan*, DiscoveRx, Fremont, CA), and the binding inhibition activity was expressed as the *K*_d_. A panel of kinases was selected, based on a previous experiment with the same assay in which regorafenib inhibited the binding of a reference compound by at least 80% at a concentration of 1 *μ*mol/L.

### Kinase assays

Pharmacologic activities of regorafenib, M‐2, and M‐5 were measured in cellular kinase phosphorylation assays. TIE‐2, wt KIT, and BRAF^V600E^ assays were performed by ProQinase (Freiburg, Germany). Cell lines used were Chinese hamster ovary (TIE‐2 assay), human acute megakaryoblastic leukemia cell line (M07e; wt KIT assay), and rat fibroblasts (RAT‐1; BRAF^V600E^ assay). VEGFR2 assays were performed in murine fibroblasts (NIH‐3T3) in the presence of 0.1 mg/mL human serum albumin. KIT^K642E^ assays were performed in a GIST cell line (GIST882) in the presence of 0.1 mg/mL BSA. Substrate phosphorylation was determined from total cell lysates by enzyme‐linked immunosorbent assay. In addition, an FGFR phosphorylation assay was performed using a human gastric carcinoma cell line (SNU‐16), with pFGFR assessed by western blot. The PDGFRA phosphorylation assay was performed by DiscoverRx using the DiscoverRx PathHunter cell line, modified to express PDGFRA. Cells were preincubated with varying concentrations of regorafenib, M‐2, or M‐5 for 60 min and subsequently stimulated with 20 ng/mL PDGF (80% maximal effective concentration) for 90–180 min. The assay signal was generated using a detection reagent cocktail and measured by chemoluminescence 13. Both the PDGFRA and FGFR assays were performed in the presence of 0.1 mg/mL BSA.

### Endothelial cell assays

Endothelial cell assays were performed as described previously 14. In brief, HuVECs and HuLECs were grown in EBM‐2 supplemented with growth factors (Lonza, Walkersville, MD). After serum starvation for 6 h in EBM‐2 containing 0.1% BSA, 2 × 10^5^ cells were treated with various concentrations of each compound for 1 h before stimulation with VEGF‐A (50 ng/mL) or VEGF‐C (200 ng/mL) for 10 min. Cells were lysed, and total cell lysates were analyzed for inhibition of phosphorylation by western blot, using antibodies against total and phosphorylated VEGFR2 and VEGFR3, ERK1/2, and AKT. Signals were detected by electrochemical luminescence (GE Healthcare Biosciences; Pittsburgh, PA).

For the migration inhibition assay, 2–3 × 10^5^ HuLECs per well were grown overnight on a gelatin‐coated 6‐well plate, serum starved for 6 h in EBM‐2 containing 0.1% BSA, and treated with regorafenib, M‐2, or M‐5 100 nmol/L for 1 h before the addition of VEGF‐C to a final concentration of 200 ng/mL. A sterile pipette tip was then used to scratch the cell layers, and images were taken after continued incubation for 40 h.

### Murine xenograft model

Mouse experiments were approved by the relevant regulatory agency of the federal state of Berlin (Landesamt für Gesundheit und Soziales Berlin, approval number G0221/03). Mice were maintained in individually ventilated cages in groups of four mice per cage. They received autoclaved food and bedding (Ssniff, Soest, Germany) and acidified (pH 4.0) tap water ad libitum. The animal facility was equipped with an automatic 12 h light/dark regulation, temperature regulation at 22 ± 2°C, and relative humidity of 50 ± 10%. Female NMRI nu/nu mice (purchased from Charles River, Sulzfeld, Germany) were inoculated with 1 × 10^7^ MDA‐MB‐231 or HT‐29 cells, which were allowed to grow subcutaneously to a palpable size of approximately 60–100 mm^3^. After random assignment, eight animals per group were administered a daily oral dose (3 mg/kg or 10 mg/kg) of regorafenib, M‐2, M‐5, or vehicle for 27 days, starting at Day 11 (HT‐29) or Day 13 (MDA‐MB‐231) after tumor inoculation. All administrations were performed in the morning, without anesthesia. Tumor volume was determined twice per week using caliper measurements, and the volume was calculated using the formula (D × d^2^)/2, with d defined as the minor axis and D as the major axis of the measurement. Tumor growth inhibition was calculated as relative tumor volume in relation to the first treatment day.

Changes in tumor volume were statistically evaluated by two‐way repeated‐measures analysis of variance, followed by Bonferroni correction, using GraphPad Prism 5 (San Diego, CA), and *P* < 0.05 was regarded as significant. Additionally, body weight of all mice was determined twice weekly, and the health status of the animals, including behavioral changes, was recorded daily.

### Pharmacokinetic studies

Pharmacokinetic studies were performed in female NMRI Foxn1 nu/nu mice, supplied by Harlan‐Winkelmann GmbH (Borchen, Germany). Regorafenib, M‐2, and M‐5 were each given to three animals per sampling time point at a dose of 10 mg/kg orally once daily for 5 days. Pharmacokinetic parameters, namely the AUC(0–24)_ss_ and *C*_max_, were calculated from plasma concentrations using a noncompartmental analysis.

### Protein‐binding analysis

The extent of binding of regorafenib and its metabolites, M‐2 and M‐5, to plasma proteins from male CD‐1 mice and white men was determined in vitro using methodology developed by Schuhmacher et al. 15. In summary, the unbound fractions of regorafenib, M‐2, and M‐5 were calculated by comparing the distribution of each compound between Transil porous silica beads (NIMBUS Biotechnology, Leipzig, Germany) and plasma with the distribution of each compound between Transil and buffer. Radiolabeled compounds ([^14^C]regorafenib, [^14^C]M‐2, and [^3^H]M‐5) were used for protein‐binding studies. A radioactivity analysis was performed by liquid scintillation counting. At least three incubations per drug concentration were performed at neutral pH and at either 37°C ([^14^C]regorafenib and [^14^C]M‐2) or room temperature ([^3^H]‐M‐5). All compounds were tested for stability at the incubation conditions used. Investigated concentrations in plasma ranged between 0.4 and 18 mg/L for regorafenib, between 0.5 and 62 mg/L for M‐2, and between 0.8 and 8 mg/L for M‐5.

## Results

### In vitro inhibition of kinase targets by M‐2 and M‐5

**Figure 1:**
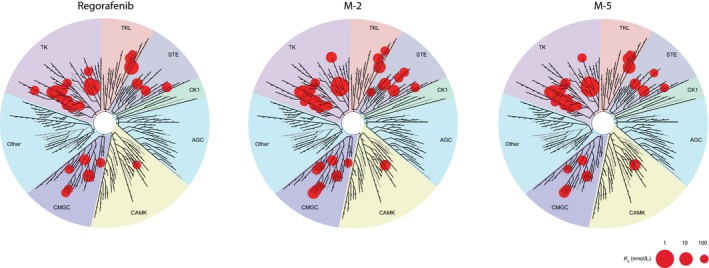
Biochemical kinase selectivity profiles of regorafenib, M‐2, and M‐5. Only kinases with *K*_d_ values ≤100 nmol/L are displayed (Table S1 for *K*_d_ values of the studied kinase panel). Image generated using the TREEspot^™^ Software Tool and reprinted with permission from KINOMEscan^®^, a division of DiscoveRx Corporation, © DiscoverRx Corporation 2010.

To assess the pharmacologic activity of M‐2 and M‐5 in comparison with the parent regorafenib compound, a competitive binding assay was performed using a selected panel of kinases for which *K*_d_ values were determined. The results demonstrate comparable kinase inhibition profiles for regorafenib, M‐2, and M‐5 (Fig. 1 and Table S1). Regorafenib, M‐2, and M‐5 inhibited key targets with similar potency, including VEGFRs (*K*_d_ values ranging from 15 to 28, 23 to 46, and 17 to 40 nmol/L, respectively, among receptor family member), KIT (*K*_d_: 6.9, 9.8, and 5.8 nmol/L, respectively), RET (*K*_d_: 5.2, 7.6, and 5.8 nmol/L, respectively), PDGFRs (*K*_d_: 8.3–19, 7.3–11, and 11 nmol/L, respectively), and RAFs (*K*_d_: 42–59, 24–130, and 11–66 nmol/L, respectively). TIE‐2 (*K*_d_: 290, 790, and 1200 nmol/L, respectively) and FGFRs (*K*_d_: 270–650, 440–880, and 790–1100 nmol/L, respectively) revealed moderate inhibition (data not shown). A number of additional kinases were inhibited with *K*_d_ values of 100 nmol/L or less, but this activity still requires confirmation in cell‐based assays (Table S1). No inhibition was observed for EGFR family kinases, the protein kinase C family, insulin and insulin‐like growth factor receptor kinases, MET, MEK family kinases (with the exception of MEK5), ERK1/2, AKT, PI3 family kinases, and ATM/ATR family kinases, even with concentrations up to 1 *μ*mol/L (data not shown).

### Cellular effects of metabolites

**Figure 2:**
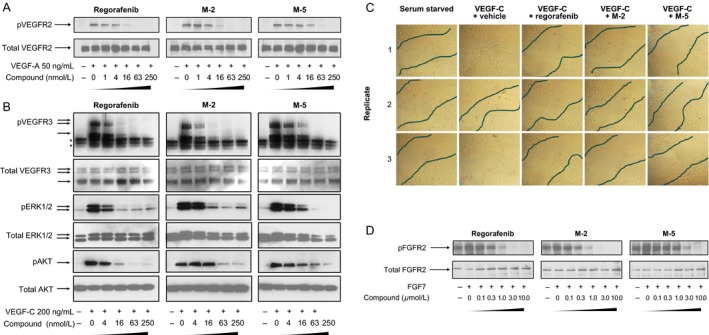
Inhibitory effects of regorafenib, M‐2, and M‐5 in cell‐based mechanistic assays. (A) VEGFR2 autophosphorylation in HuVECs. (B) VEGFR3 autophosphorylation in HuLECs and effects on potential intracellular signaling kinases ERK1/2 and AKT. *Indicates unspecific signals. (C) Inhibition of cell migration, analyzed by scratch assay in HuLECs. Black lines demarcate the borders of the confluent cell layer. (D) Inhibition of FGFR2 autophosphorylation in SNU‐16 tumor cells. Regorafenib data in A, B, and C were taken from Schmieder et al. 14.

To further assess the pharmacologic potency of M‐2 and M‐5, their inhibition of target kinases by regorafenib was investigated in cell‐based assays. The antiangiogenic effects of M‐2 and M‐5 were compared with those of regorafenib by investigating VEGFR2 and VEGFR3, both of which play key roles in angiogenesis and lymphangiogenesis. M‐2 and M‐5, both potently inhibited VEGFR2 and VEGFR3 autophosphorylation in serum‐deprived HuVECs and HuLECs following stimulation with VEGF‐A and VEGF‐C, respectively (Fig. 2A and B) 14. The IC_50_ values for M‐2 and M‐5, estimated from western blots, were similar to that of regorafenib for both receptors, with M‐5 being slightly less effective (~4−16 nmol/L; Table 1). Total protein amounts of VEGFR2 and VEGFR3 were unchanged (Fig. 2A and B).

**Table 1:** Cellular kinase assays of regorafenib, M‐2, and M‐5

Kinase target	Mean ± SD IC_50_ (nmol/L)			Cell line
	Regorafenib	M‐2	M‐5	
BRAF^V600E^	69	21	27	RAT‐1/ BRAF^V600E^ a
FGFR2	~200	~200	~1000	SNU‐16/FGFR2^b^
KIT	23	13	110	M07e/KIT^a^
KIT^K642E^ c	17 ± 4 (2)	4 ± 2 (2)	nd	GIST882/KIT^K642E^ b
PDGFRα	136	44	61	U2OS/PDGFRA^b^
TIE‐2^c^	31 ± 9 (3)	66 ± 35 (2)	180 ± 0 (2)	CHO/TIE2^a^
VEGFR2	~4	~4	~16	HuVECs^b^
VEGFR3	~4	~4	~16	HuLECs^b^

a Without serum.

b Assay performed in 0.1% bovine serum albumin.

c Numbers in brackets refer to the number of experiments.

CHO, Chinese hamster ovary; HuLECs, human lymphatic endothelial cells; HuVECs, human vascular endothelial cells; IC50, half maximal inhibitory concentration; nd, not determined; SD, standard deviation.

Regorafenib, M‐2, and M‐5 inhibited the activation of ERK and AKT kinases in HuLECs with nanomolar IC_50_ values similar to those for VEGFR3 (4–16 nmol/L; Fig. 2B). Total protein amounts of ERK1/2 and AKT were minimally affected (see Fig. 2B). M‐2 and M‐5 also inhibited VEGFR2 activity with higher potency than regorafenib in a kinase phosphorylation assay using NIH‐3T3 cells (data not shown).

Regorafenib, M‐2, and M‐5—all inhibited VEGF‐C‐induced migration of HuLECs (Fig. 2C). No apoptotic cells were detected during the assay, despite serum deprivation and potent inhibition of the AKT pathway.

Along with key angiogenic kinases, we tested the activity of M‐2 and M‐5 on selected oncogenic kinases, mutant and wtKIT, and mutant BRAF. Cellular kinase assays demonstrated that M‐2 and M‐5 inhibited key regorafenib targets, with IC_50_ values in a similar nanomolar range to those of regorafenib. Indeed, M‐2 inhibited all targets, except TIE‐2, with IC_50_ values comparable to or lower than those of regorafenib. Inhibition of mutant KIT^K642E^ was not determined with M‐5 (Table 1).

The activity of regorafenib, M‐2, and M‐5 at PDGFRα and FGFR2, which are associated with maintenance of the tumor microenvironment 16, was also assessed in cell‐based assays. In the PDGFRA assay—regorafenib, M‐2, and M‐5—all showed inhibitory activity, with M‐2 and M‐5 both showing greater potency than regorafenib (Table 1). Regorafenib and M‐2 also showed some activity at FGFR2, with IC_50_ values of approximately 0.2 *μ*mol/L, whereas the IC_50_ for M‐5 at FGFR2 was about 1 *μ*mol/L (Fig. 2D and Table 1).

### Antitumor activity in murine xenograft models

**Figure 3:**
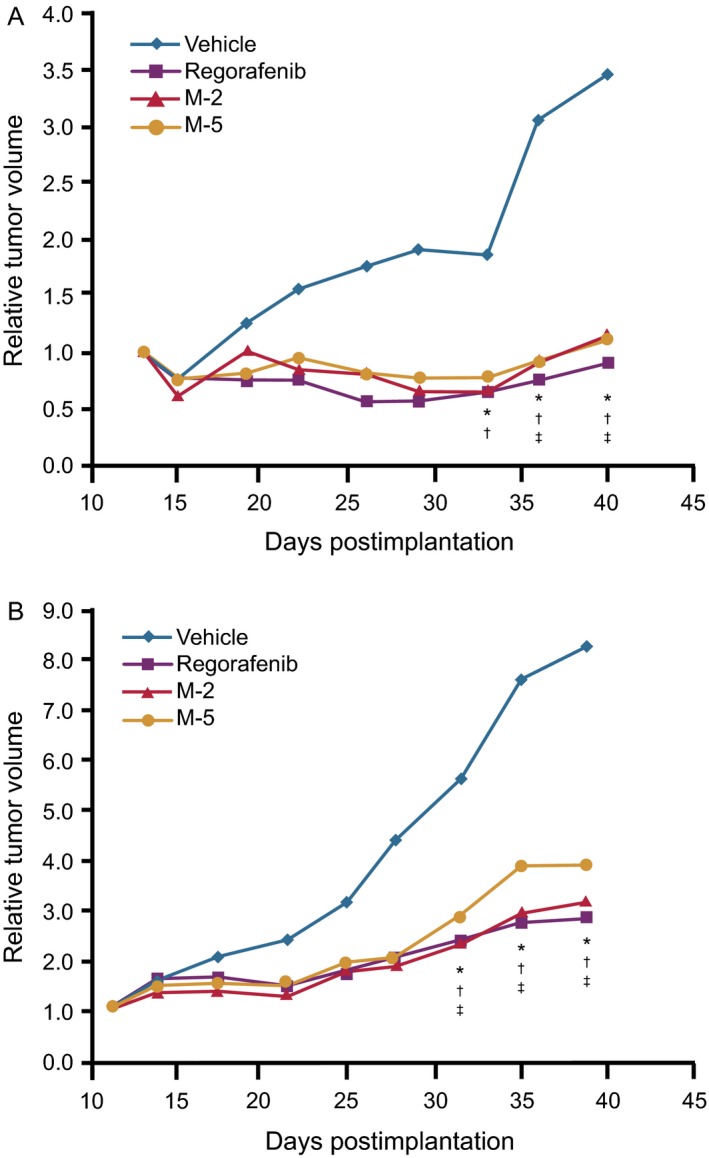
Effects of regorafenib, M‐2, and M‐5 on the growth of human xenografts in mice. Data show relative tumor volume in mice bearing xenografts of (A) human breast cancer cell line MDA‐MB‐231 (KRAS^G13D^, BRAF^G464V^) and (B) human CRC cell line HT‐29 (BRAF^V600E^) following oral administration of 10 mg/kg/day of regorafenib, M‐2, or M‐5 for 27 days, starting at Day 13 or Day 11, respectively, after tumor inoculation (palpable tumor size; *n *=* *8; *P <* *0.05 for regorafenib versus vehicle; ^†^ *P *<* *0.05 for M‐2 versus vehicle; ^‡^ *P *<* *0.05 for M‐5 versus vehicle)

The dose‐dependent in vivo activity of M‐2 and M‐5 in HT‐29 colon cancer and MDA‐MB‐231 breast cancer xenograft models was investigated in NMRI nu/nu mice—a strain that is genetically similar to the one used in the pharmacokinetic analyses. These studies demonstrated that M‐2 and M‐5 caused potent inhibition of tumor growth at 10 mg/kg/day, similar to that observed with the regorafenib parent compound at the 10 mg/kg/day dose (Fig. 3, Table S3). In the MDA‐MB‐231 model, previously shown to be particularly responsive to regorafenib 1, regorafenib, M‐2, and M‐5 all significantly inhibited tumor growth relative to a vehicle control group (*P *<* *0.05 for all groups). Similarly, all agents showed significant inhibition over vehicle controls in the HT‐29 model (*P *< 0.05 for all groups), but generally at a slightly lower range than that observed in the MDA‐MB‐231 model. The antitumor effects of 3 mg/kg/day doses of regorafenib, M‐2, and M‐5 were also investigated in the two xenograft models (Fig. S2; Table S2). Tumor growth inhibition was similar for regorafenib and M‐2 at the 3 mg/kg/day dose in both models, but with lower inhibition than the 10 mg/kg/day dose. M‐5 showed less growth inhibition at the 3 mg/kg/day dose in the HT‐29 model than the other agents, but higher inhibition in the MDA‐MB‐231 model (similar inhibition to the 10 mg/kg/day dose). All treatments were well tolerated, with body weight losses below 5% and no signs of adverse effects.

### Pharmacokinetic studies

To mimic exposure to regorafenib in preclinical pharmacology studies, the plasma pharmacokinetics of regorafenib and its metabolites were investigated in female NMRI Foxn1 nu/nu mice after repeated oral administration of 10 mg/kg of parent regorafenib, M‐2, or M‐5 for 5 consecutive days to achieve steady‐state levels. In regorafenib‐treated mice, the sum of the total AUC(0–24)_ss_ of regorafenib and its active major human metabolites was 46,911 μg × h/L, with the majority of exposure attributed to regorafenib (82%), while M‐2 and M‐5 accounted for 16% and 2% of total exposure, respectively (Table 2). A *C*_max_ of 4146 *μ*g/L was measured for regorafenib (administered compound), whereas the *C*_max_ of in vivo formed metabolites M‐2 and M‐5 reached only 753 *μ*g/L and 63 *μ*g/L, respectively, resulting in an overall total *C*_max_ for all three compounds of about 5000 *μ*g/L (Table 2).

**Table 2:** Pharmacokinetic parameters of regorafenib and its metabolites M‐2 and M‐5 in NMRI Foxn1 nu/nu mice after oral administration at a dose of 10 mg/kg/day for 5 consecutive days

Administered compound	Regorafenib			M‐2			M‐5
Analyte	Regorafenib	M‐2	M‐5	Regorafenib	M‐2	M‐5	M‐5^a^
AUC (*μ*g × h/L)	38,649	7490	772	11,601	52,101	3391	52,796
AUC (*μ*Mol × h/L)	80.0	15.0	1.6	24.0	104.4	7.0	108.9
AUC (% of total)^b^	83	16	2	18	78	5	100
*C*_max_ (*μ*g/L)	4146	753	63	967	6150	342	5284
*C*_max_ (*μ*M/L)	8.6	1.5	0.1	2.0	12.3	0.7	10.9

a Only M‐5 concentration was determined, because M‐2 and regorafenib formation does not occur.

b Total refers to the sum of the molar concentrations of regorafenib, M‐2, and M‐5 regardless of unbound fraction.

Administration of M‐2 resulted in a sum of total AUC(0–24)_ss_ of 67,093 *μ*g × h/L, with M‐2 accounting for 78%, regorafenib (presumably formed by reduction of M‐2 in the intestinal milieu) for 17%, and M‐5 for 5% of total exposure (Table 2). The sum of regorafenib, M‐2, and M‐5 reached an overall total *C*_max_ of about 7500 *μ*g/L, with the highest contribution of 6150 *μ*g/L by M‐2, and regorafenib and M‐5 accounting for 967 *μ*g/L and 342 *μ*g/L, respectively.

Administration of M‐5 resulted in an AUC(0–24)_ss_ of 52,796 *μ*g × h/L and a *C*_max_ of approximately 5300 *μ*g/L (Table 2).

### Plasma protein binding

The pharmacologic activity of small‐molecule compounds, in vitro and in vivo, is strongly dependent on their binding to plasma proteins 17, 18. Therefore, we analyzed the binding of regorafenib, M‐2, and M‐5 to murine and human plasma in vitro (Table 3). Overall, the protein binding for all three compounds was high in both species, with unbound fractions below 1%. The binding of M‐5 to human plasma proteins was most pronounced, with an unbound fraction of ~0.05%—approximately 10‐fold lower than that of regorafenib (~0.5%). Binding of M‐2 to human plasma proteins was also higher than that of the parent drug, with an unbound fraction of ~0.2%, about 2.5‐fold lower than that of regorafenib. In contrast, significantly smaller differences in plasma protein binding were observed for murine plasma, with the unbound fraction of M‐2 (~0.9%) approximately 1.5‐fold higher than that of regorafenib (~0.6%), while the unbound fraction of M‐5 was ~0.4%. Furthermore, although the unbound fraction of regorafenib was similar in murine and human plasma, the unbound fractions of M‐2 and M‐5 were about fivefold and eightfold higher, respectively, in murine plasma than in human plasma. The major plasma protein to which regorafenib, M‐2, and M‐5 were bound was albumin (data not shown) as tested in human plasma.

**Table 3:** Plasma protein binding of regorafenib, M‐2, and M‐5

Compound	Unbound fraction (%)	
	Mouse,^a^^,^ b male	Human,^c^ male
Regorafenib	0.575 ± 0.039	0.488 ± 0.091
M‐2	0.888 ± 0.033	0.188 ± 0.004
M‐5	0.412 ± 0.041	0.053 ± 0.013

Data are arithmetic means ± standard deviations.

a Protein binding was assessed in CD‐1 mice.

b Regorafenib *n *=* *4; M‐2 *n *=* *2; M‐5 *n *=* *11–14.

c Regorafenib *n *=* *4; M‐2 *n *=* *6; M‐5 *n *=* *11–14.

## Discussion

Clinical trials have shown that total steady‐state exposure to the M‐2 and M‐5 metabolites of regorafenib in humans is similar to that of the parent drug 4, 5, raising the possibility that these metabolites may contribute to the observed clinical activity after oral administration of regorafenib. However, the activity of M‐2 and M‐5 cannot be definitely evaluated in humans. We observed that the biochemical and antitumor activities of M‐2 and M‐5 are similar to those of the regorafenib parent compound in our preclinical models, indicating the potential of M‐2 and M‐5 to contribute to the overall clinical efficacy of regorafenib.

Combined data from biochemical and cellular assays, as well as preclinical in vivo studies, indicate that M‐2 and M‐5 show similar pharmacologic activity to that of the parent compound, with both metabolites demonstrating similar kinase inhibition activity and selectivity profiles to those of regorafenib. The biochemical kinase activity results were generated by a competitive binding assay 12 and are consistent with previously reported enzymatic assays 1, indicating that the two assay types deliver comparable results. Differences between competitive binding assays and enzymatic assays in the measured *K*_d_ of regorafenib at VEGFR2 and RAF‐1 might be attributable to interspecies differences, variations in the protein preparation, or variations in assay conditions.

Regorafenib was found to potently inhibit a number of mutant and wt kinases in biochemical assays, including its previously identified key target kinases, with *K*_d_ values below 100 nmol/L (Table S1). Some of these kinases have been implicated in cancer, such as mutant forms of the receptor tyrosine kinases FLT3 and DDR2, which have been identified in acute myeloid leukemia and lung cancer, respectively 19, 20. Such kinases potentially provide new opportunities for the therapeutic use of regorafenib. However, our own preliminary in vitro proliferation experiments using lung tumor cell lines expressing wt or mutant DDR2 showed only moderate inhibition by regorafenib, M‐2, and M‐5 compared with potent inhibition of BaF/3 cells expressing an imatinib‐resistant mutant KIT (557‐558del; T670I) by regorafenib (unpubl. data). This strongly indicates that additional studies are required to assess the potential value of potent biochemical inhibition of these kinases by regorafenib.

Neither regorafenib nor M‐2 and M‐5 significantly inhibit ERK1/2 and AKT in biochemical assays (data not shown); therefore, the observed reductions of pERK1/2 and pAKT levels in cell‐based assays are presumably indirect effects due to the inhibition of kinases located upstream in their signaling pathway, such as VEGFR3 and Raf.

Cellular kinase assay data for regorafenib‐mediated inhibition of VEGFR2 and FGFR2 autophosphorylation show comparable IC_50_ values to those previously reported 1, 14. However, the regorafenib‐mediated inhibition of VEGFR3 autophosphorylation in this study appears more potent than reported previously 1, which may be related to species differences or the cellular context. In this study, growth factor‐induced autophosphorylation of VEGFR2 and VEGFR3 was inhibited by regorafenib, M‐2, and M‐5 in HuVECs and HuLECs. The IC_50_ range for the inhibition of VEGFR2 phosphorylation in biochemical assays was similar to that observed in cellular kinase assays, indicating comparability between these two analyses. The antilymphangiogenic properties of M‐2 and M‐5 were further demonstrated in a cell migration assay, showing a similar effect to that of regorafenib. In addition, it is likely that the FGFR1 assay in SNU‐16 cells was detecting FGFR2 activity because FGF7 (the ligand used in the assay) has previously been reported to be specific for FGFR2 21. Furthermore, FGFR2, but not FGFR1, is overexpressed in the SNU‐16 cell line 22. Although all cell‐based assays reported here were performed at low albumin concentrations (mostly BSA) or in the absence of serum in order to avoid protein‐binding effects, it should be mentioned that mimicking of protein‐binding effects in in vitro assays has been difficult 17, 18, 23. In addition, variation in protein binding between species, demonstrated here with murine and human plasma, precludes extrapolation of protein‐binding data to in vitro cell cultures performed using other common protein sources, such as fetal calf serum.

In vivo, tumor xenograft studies showed that the M‐2 and M‐5 metabolites both had similar antitumor activity to that of the parent compound. This finding is consistent with the in vitro data showing the generally similar ability of the three compounds to inhibit the target kinases, as well as the pharmacokinetic data for all three compounds demonstrating comparable protein binding, total exposure (sum of AUCs), and *C*_max_ in mice. The originally administered compound accounted for the majority of the total AUC and *C*_max_, consistent with the proposed biotransformation pathway of regorafenib in humans shown in Figure S1, with a reversible biotransformation leading to the formation of the M‐2 metabolite from regorafenib and vice versa. However, formation of the M‐5 metabolite from M‐2 results from a nonreversible biotransformation due to demethylation.

To assess antitumor activity in murine models and perhaps improve the usefulness of these models for predicting activity in humans, we analyzed various pharmacokinetic parameters and the protein binding of regorafenib and its metabolites M‐2 and M‐5. Notably, regorafenib exposure and total *C*_max_ plasma concentrations in mice after once‐daily dosing with regorafenib 10 mg/kg for 5 days were comparable to those in humans receiving 160 mg/day in a 3 weeks on/1 week off dose regimen, which was defined as the maximum tolerated dose 4. Although present at lower total steady‐state exposures than regorafenib, metabolites M‐2 and M‐5 were detected in the plasma of mice, suggesting that the oxidative biotransformation pathway of regorafenib is similar in humans 4, 5 and mice.

Another essential factor for the assessment of the pharmacologic activity of small‐molecule compounds is the proportion bound to biologic molecules, in particular, plasma and tissue proteins. A high proportion of protein‐bound drug (more than 99%) was observed for regorafenib, M‐2, and M‐5 in both murine and human plasma. Although the unbound fraction of regorafenib was similar in murine and human plasma, marked interspecies differences were observed in the unbound fractions of M‐2 and M‐5, with ~5‐fold and ~8‐fold lower unbound fractions, respectively, in human than in murine plasma.

To relate the unbound fraction with in vivo pharmacological activity, it is important to consider that total drug concentration is only a relative value, and that the free drug concentration is the more relevant measure. This allows comparison of the free plasma drug concentration with the IC_50_ values of the target kinases determined in biochemical or cell‐based assays, providing an estimate of the contribution of each compound to the in vivo pharmacologic activity of regorafenib. The calculated maximum free drug concentrations for regorafenib, M‐2, and M‐5 were 49.4, 13.4, and 0.5 nmol/L, respectively, in murine plasma. These closely matched the free drug concentrations for regorafenib, M‐2, and M‐5 of 42.1, 13.5, and 3.4 nmol/L, respectively, in human plasma. These concentrations are well above the IC_50_ values of approximately 4 nmol/L for regorafenib and M‐2 for human VEGFR2 in HuVECs demonstrated in this study, as well as the IC_50_ values of approximately 4 nmol/L for recombinant murine VEGFR2 1. In contrast, concentrations of M‐5 appear to be distinctly below the determined IC_50_ value of approximately 16 nmol/L for human VEGFR2, which suggests that M‐5 may have a limited contribution to the overall efficacy of regorafenib, despite the similarity of its pharmacologic profile to parent regorafenib. Ultimately, pharmacokinetic and pharmacodynamic analyses of tumor tissue that provide an integrated read‐out are required in order to determine the kinetics of local target kinase inhibition.

In this study, in vitro experiments and in vivo studies in mice were used to assess the pharmacologic activity of the major human metabolites of regorafenib and extensive pharmacokinetic analyses were performed to provide an estimate for their contribution to their therapeutic efficacy in tumor patients. Although the compounds show behavioral similarities between species, such as the oxidative biotransformation of the parent compound regorafenib, variabilities were observed in other cases such as protein binding. Considering that physiology is more complex, predictions across species have, therefore, to be taken with some caution.

## Conclusion

Overall, these preclinical data show that the biochemical and antitumor activities of M‐2 and M‐5 are similar to those of the regorafenib parent compound. When the estimated in vivo free plasma concentrations of regorafenib and its metabolites are taken into consideration, the data suggest that M‐2, but not M‐5, has a high likelihood of contributing to the overall efficacy of regorafenib.

## Conflict of Interest

DZ, WS, KD, SW, KHT, FT‐H, and MG are full‐time employees of Bayer Pharma AG. IF is the chief scientific officer of Experimental Pharmacology & Oncology GmbH. AB is the chief scientific officer of Shakti BioResearch LLC.

## Supporting information


**Figure S1.** Oxidative biotransformation pathways of regorafenib^10^.
**Figure S2.** Effects of regorafenib and its metabolites M‐2 and M‐5 on the growth of human tumor xenografts in mice.
**Table S1.** Biochemical kinase selectivity profiles of regorafenib, M‐2, and M‐5. Part *a* lists kinases with *K*_d_ values ≤ 100 nmol/L for at least one compound.
**Table S2.** Growth inhibition of human colorectal cancer (HT‐29) and breast cancer (MDA‐MB‐231) xenografts in mice by regorafenib, M‐2, and M‐5.
