# Supplementary material for: Pharmacologic activity and pharmacokinetics of metabolites of regorafenib in preclinical models
Source: Cancer Med. 2016 Oct 13;5(11):3176–85. doi: 10.1002/cam4.883 (PMC5119973; doi:10.1002/cam4.883)
Supplement: Supplementary file 1 — Figure S1. Oxidative biotransformation pathways of regorafenib10. Figure S2. Effects of regorafenib and its metabolites M‐2 and M‐5 on the growth of human tumor xenografts in mice. Table S1. Biochemical kinase selectivity profiles of regorafenib, M‐2, and M‐5. Part a lists kinases with K d values ≤ 100 nmol/L for at least one compound. Table S2. Growth inhibition of human colorectal cancer (HT‐29) and breast cancer (MDA‐MB‐231) xenografts in mice by regorafenib, M‐2, and M‐5. [file CAM4-5-3176-s001.docx]

Pharmacologic activity and pharmacokinetics of metabolites of regorafenib in preclinical models

Dieter Zopf^1^, Iduna Fichtner^2^, Ajay Bhargava^3^, Wolfram Steinke^1^, Karl-Heinz Thierauch^1^,
Konstanze Diefenbach^1^, Scott Wilhelm^4^, Frank-Thorsten Hafner^1^, Michael Gerisch^1^

^1^Global Drug Discovery, Bayer Pharma AG, Germany
^2^Experimental Pharmacology & Oncology GmbH, Berlin-Buch Germany
^3^Shakti Bioresearch, Hamden, CT, USA
^4^Bayer HealthCare Pharmaceuticals, Whippany, NJ, USA

**Supplementary data**

**Supplementary Figure S1.** Oxidative biotransformation pathways of regorafenib^10^ **
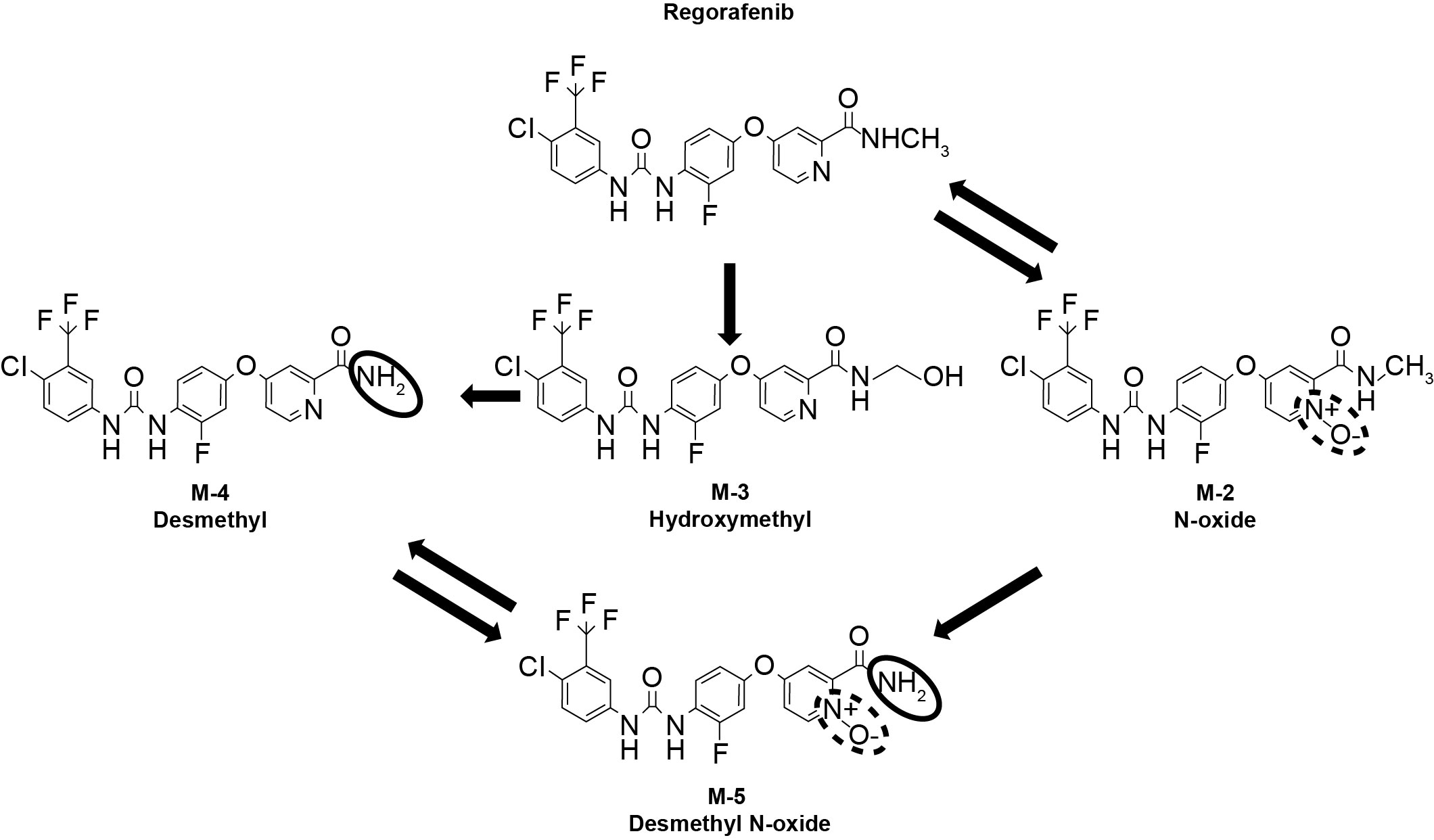
**

**Supplementary Figure S2.** Effects of regorafenib and its metabolites M-2 and M-5 on the growth of human tumor xenografts in mice. Data show relative tumor volume in mice bearing xenografts of (*a*) human breast cancer cell line MDA-MB-231 (*KRAS*^G13D^, *BRAF*^G464V^) and (*b*) human colorectal cancer cell line HT-29 (*BRAF*^V600E^) following oral administration of 3 mg/kg/day of regorafenib, M-2, or M-5 for 27 days, starting at Day 13 or Day 11, respectively, after tumor inoculation (palpable tumor size; *n* = 8; **p* < 0.05 for regorafenib *vs* vehicle; ^†^*p* < 0.05 for M-2 *vs* vehicle; ^‡^*p* < 0.05 for M-5 *vs* vehicle).


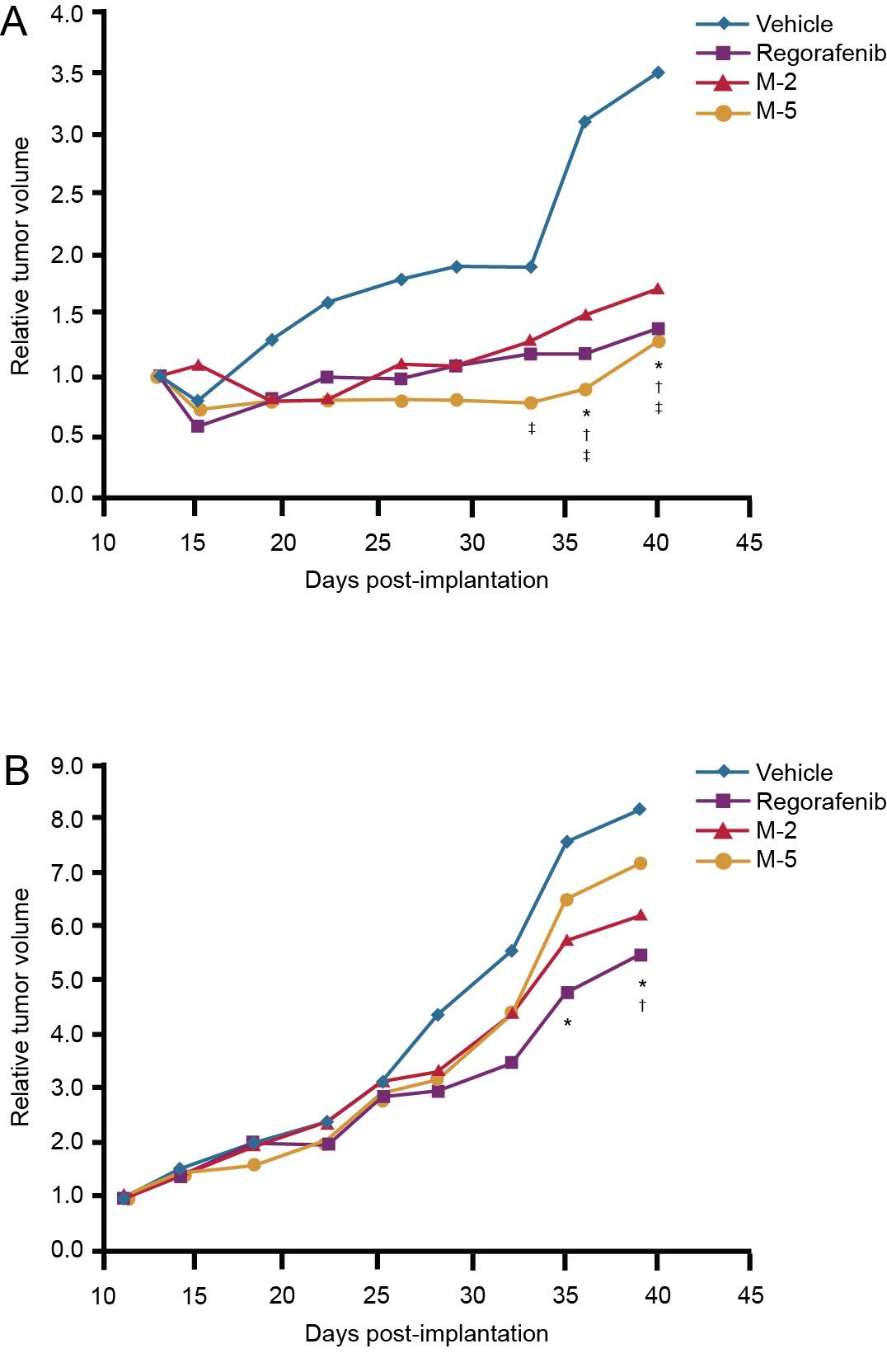


**Supplementary Table 1.** Biochemical kinase selectivity profiles of regorafenib, M-2, and M-5. Part *a* lists kinases with *K*_d_ values ≤100 nmol/l for at least one compound. Part *b* lists *K*_d_ values for all mutant kinases tested irrespective of *K*_d_ value. Duplicate *K*_d_ values are given where available. Numbers in bold were derived from the same assay and are used in Figure 1.

| ***a*** | ***K*_d_ (nmol/l)** | | |
| --- | --- | --- | --- |
| **Compound/gene symbol** | **Regorafenib^*^** | **M-2** | **M-5** |
| ABL1-np | **32**/16 | **23** | **20** |
| BRAF | **42**/52 | **24** | **17** |
| CDK11 | **67**/43 | **33** | **86** |
| CDK8 | **100**/73 | **43** | **140** |
| CDKL2 | **48**/58 | **76** | **1100** |
| CSF1R | **43**/10 | **21** | **13** |
| DDR1 | **0.9**/0.8 | **0.5** | **0.4** |
| DDR2 | **9.2**/9.7 | **5.3** | **2.5** |
| EPHA6 | **48**/23 | **210** | **640** |
| EPHA8 | **140**/130 | **61** | **73** |
| ERK8 | **25**/17 | **16** | **26** |
| FLT1 | **28**/27 | **23** | **17** |
| FLT3 | **9.6**/4.8 | **6.7** | **2.6** |
| FLT4 | **41**/15 | **46** | **40** |
| FRK | **69**/42 | **42** | **24** |
| HIPK4 | **6.9**/4.5 | **15** | **26** |
| JNK2 | **1,400**/1,900 | **67**/44 | **670** |
| KIT | **35**/6.9 | **9.8** | **5.8** |
| LOK | **15**/10 | **9.3** | **11** |
| MAP4K4 | **450**/400 | **42** | **89** |
| MEK5 | **40** | **10** | **10** |
| MKNK2 | **90**/64 | **20** | **11** |
| MUSK | **68**/67 | **140** | **140** |
| p38-alpha | **58**/48 | **17** | **48** |
| p38-beta | **44**/28 | **8.7** | **25** |
| PDGFRA | **21**/19 | **7.3** | **11** |
| PDGFRB | **19**/8.3 | **11** | **11** |
| RAF1 | **87**/59 | **130** | **66** |
| RET | **7.7**/5.2 | **7.6** | **5.8** |
| SLK | **120**/98 | **64** | **100** |
| TAK1 | **170**/260 | **79** | **120** |
| TAOK3 | **200**/290 | **94**/77 | **450** |
| TIE-1 | **30**/27 | **48** | **73** |
| TNIK | **1,100**/970 | **41** | **120** |
| TNNI3K | **170**/110 | **160** | **77** |
| TRKC | **180**/280 | **15** | **56** |
| VEGFR2 | **57**/28 | **29** | **31** |
| YSK4 | **38**/94 | **55** | **70** |
| ZAK | **2.8**/2 | **6.7** | **7** |

| ***b*** | ***K*_d_ (nmol/l)** | | |
| --- | --- | --- | --- |
| **Compound/gene symbol** | **Regorafenib** | **M-2** | **M-5** |
| BRAF^V600E^ | 30 | 15 | 11 |
| FLT3^D835H^ | 68 | 120 | 120 |
| FLT3^ITD^ | 36 | 43 | 19 |
| FLT3^K663Q^ | 7.7 | 10 | 5.5 |
| FLT3^N841I^ | 24 | 34 | 23 |
| FLT3^R834Q^ | 200 | 240 | 270 |
| KIT^A829P^ | 23 | 19 | 19 |
| KIT^D816H^ | 840 | 490 | 460 |
| KIT^D816V^ | 370 | 300 | 310 |
| KIT^L576P^ | 16 | 17 | 9.1 |
| KIT^V559D^ | 15 | 12 | 9.6 |
| KIT^V559D,T670I^ | 13 | 12 | 11 |
| KIT^V559D,V654A^ | 65 | 51 | 42 |
| RET^M918T^ | 7.1 | 7.4 | 5.8 |
| RET^V804L^ | 19 | 13 | 15 |
| RET^V804M^ | 23 | 20 | 25 |

^*^*K*_d_ values reported for regorafenib represent the results of two separate measurements.

np: not phosphorylated.

**Supplementary Table 2:** Growth inhibition of human colorectal cancer (HT-29) and breast cancer (MDA‑MB-231) xenografts in mice by regorafenib, M-2, and M-5

|  | **Dose (mg/kg/day)** | **Tumor growth inhibition (%)** | |
| --- | --- | --- | --- |
| **Compound administered** |  | **HT-29** | **MDA-MB-231** |
| Regorafenib | 3 | 34 | 44 |
|  | 10 | 67 | 72 |
| M-2 | 3 | 35 | 49 |
|  | 10 | 59 | 70 |
| M-5 | 3 | 13 | 60 |
|  | 10 | 66 | 59 |

Data are based on mean values at the end of the study (day 39 for HT-29 and day 40 for MDA-MB-231).
